# DUF1220 copy number is associated with schizophrenia risk and severity: implications for understanding autism and schizophrenia as related diseases

**DOI:** 10.1038/tp.2015.192

**Published:** 2015-12-15

**Authors:** V B Searles Quick, J M Davis, A Olincy, J M Sikela

**Affiliations:** 1 Department of Biochemistry and Molecular Genetics, Human Medical Genetics and Genomics and Medical Scientist Training Programs, University of Colorado Anschutz Medical Campus, Aurora, CO, USA; 2 Department of Psychiatry, University of Colorado Anschutz Medical Campus, Aurora, CO, USA

## Abstract

The copy number of DUF1220, a protein domain implicated in human brain evolution, has been linearly associated with autism severity. Given the possibility that autism and schizophrenia are related disorders, the present study examined DUF1220 copy number variation in schizophrenia severity. There are notable similarities between autism symptoms and schizophrenia negative symptoms, and divergence between autism symptoms and schizophrenia positive symptoms. We therefore also examined DUF1220 copy number in schizophrenia subgroups defined by negative and positive symptom features, versus autistic individuals and controls. In the schizophrenic population (*N*=609), decreased DUF1220 copy number was linearly associated with increasing positive symptom severity (CON1 *P*=0.013, HLS1 *P*=0.0227), an association greatest in adult-onset schizophrenia (CON1 *P*=0.00155, HLS1 *P*=0.00361). In schizophrenic males, DUF1220 CON1 subtype copy number increase was associated with increased negative symptom severity (*P*=0.0327), a finding similar to that seen in autistic populations. Subgroup analyses demonstrated that schizophrenic individuals with predominantly positive symptoms exhibited reduced CON1 copy number compared with both controls (*P*=0.0237) and schizophrenic individuals with predominantly negative symptoms (*P*=0.0068). These findings support the view that (1) autism and schizophrenia exhibit both opposing and partially overlapping phenotypes and may represent a disease continuum, (2) variation in DUF1220 copy number contributes to schizophrenia disease risk and to the severity of both disorders, and (3) schizophrenia and autism may be, in part, a harmful by-product of the rapid and extreme evolutionary increase in DUF1220 copy number in the human species.

## Introduction

Schizophrenia is a complex neuropsychiatric disease that is estimated to affect up to 1% of the global population.^1^ Schizophrenic individuals exhibit a wide range of phenotypic heterogeneity, with symptoms often broadly grouped into positive and negative categories.^2, 3^ Positive symptoms include gain of features not present in healthy individuals, such as hallucinations and delusions, whereas negative symptoms are characterized by loss of features present in healthy individuals, producing symptoms such as flattened affect, anhedonia, and decreased social capacities. Schizophrenia as a whole exhibits complex genetic and neurodevelopmental etiologies that are poorly understood.^4^ Although the heritability of schizophrenia is estimated to be upwards of 80%,^5^ genome-wide studies have been able to explain only a small portion of the disease.^6, 7^ There has been a great deal of discussion surrounding this ‘missing heritability', and it has been theorized that a large portion of such heritability may lie in previously unexplored regions of the genome that have been ignored in genotype−phenotype analyses owing to assay limitations.^8^

DUF1220 is a protein domain encoded almost exclusively by genes of the neuroblastoma breakpoint family (*NBPF)* on chromosome 1q21.^9, 10, 11^ This region has repeatedly been shown to harbor copy number variations (CNVs) that are associated with autism and schizophrenia^12, 13, 14, 15^ and that encompass many DUF1220 copies.^9^ Interestingly, 1q21-associated duplications are enriched in individuals with autism and reciprocal deletions are enriched in those with schizophrenia.^16, 17^ DUF1220 domains can be subdivided into six different subtypes, or clades, based on sequence similarity: conserved (CON) clades 1, 2 and 3, and human lineage-specific (HLS) clades 1, 2 and 3.^18^ The copy number of DUF1220 has increased dramatically during primate evolution, with the most extreme increase found in the human lineage.^11^ Notably, the unique increase in copy number seen in humans compared with chimpanzees (290 versus 125) is due predominantly to an increase in the copy number of the HLS subtypes.^18, 19^ This evolutionary increase in copy number has been linearly associated with an evolutionary increase in brain size and cortical neuron number, suggesting an evolutionary advantage associated with copy number expansion of the domain.^20, 21^ Analyses of functional effects of CNV in human populations have shown that DUF1220 copy number is significantly associated with both healthy and pathogenic brain size variation,^21^ cognitive ability^8^ and severity of symptoms in autism.^22, 23^

Multiple studies have suggested that schizophrenia and autism are related disorders.^17, 24, 25^ The predominant theories are that they are (1) overlapping disorders, (2) subtypes of the same disease, or (3) diametric opposites (or entirely separate disorders; for an overview of theories see ref. 26). Both the overlapping theory and diametric opposites theory could be considered in the context of a continuum, such that the two diseases are related on one disease spectrum. The location of each disease on this spectrum varies based on the theory in question. Evidence is available to support each of these propositions, and it is plausible that the true nature of the relationship is more nuanced and explained by more than one of these theories. With respect to overlapping and subtype hypotheses, the primary phenotypic evidence cited is that the negative features of schizophrenia (e.g., asociality and introversion) are similar to the symptoms of autism (e.g., communication and social deficits).^24, 27^ The diametric opposites theory, meanwhile, is supported by the divergent characteristics of positive symptoms of schizophrenia (such as hallucinations and delusions) versus symptoms of autism.

One of the most striking and well-documented genomic findings suggests that schizophrenia and autism may be diametric opposites. CNVs in at least four different genomic regions (1q21, 16p11.2, 22q11.21 and 22q13.3) have been identified in which duplications are significantly associated with one disorder while reciprocal deletions are associated with the other (reviewed in ref. 26). With respect to 1q21, duplications are more frequently involved in autism, and reciprocal deletions more frequently involved in schizophrenia.^28, 29^ Likewise, some disease phenotypes and neuropathologies exhibit inverse relationships between the two disorders. Autistic individuals often exhibit abnormally increased brain growth and in turn relatively increased brain size compared with controls,^30, 31^ while schizophrenic individuals often have reduced brain volumes.^32, 33^ Such findings imply that the dosage of key sequences within these CNVs contribute to both schizophrenia and autism but in opposite directions.^9^

Recent studies have shown that DUF1220 copy number is linearly and positively associated with autism severity.^22, 23^ Given these findings, that many DUF1220 domain copies reside within the autism- and schizophrenia-associated 1q21 region, and that DUF1220 copy number is associated with brain size variation, the present study sought to determine whether DUF1220 is associated with features of schizophrenia. Specifically, we sought to determine (1) whether DUF1220 CNV is associated with positive and negative symptoms of schizophrenia, (2) whether copy number differs between schizophrenic individuals and controls, and (3) whether copy number differs between schizophrenic individuals and autistic individuals. In addition, we stratified the schizophrenic population into groups with predominantly positive or predominantly negative symptoms, similar to previous studies.^34, 35, 36^ This was performed based on aforementioned work suggesting that the two symptom types are associated with unique neurobiologic and physiologic features and the purported similarity between negative symptoms and symptoms of autism. We hypothesized that (1) in the predominantly negative symptom group, due to its similarity to autism, there would be a similar linear association between negative symptom severity and DUF1220 copy number (as seen for symptom severity in individuals with autism), and (2) this same association would not be found in the predominantly positive symptom group.

## Materials and Methods

### Sample selection

DNA from 609 individuals diagnosed with schizophrenia using DSM-IV criteria was selected from the NIMH Repository and Genomics Resource and acquired from the cell and DNA repository at Rutgers University, along with 120 controls with no known diagnoses and 168 individuals with autism spectrum disorder (ASD). All individuals were non-Hispanic white to control for population stratification. Power was assumed to be adequate based on effect size and variance from previous work in autistic populations.^22, 23^ Individuals with ASD are consenting participants in the Autism Genetic Resource Exchange. Individuals with schizophrenia and otherwise healthy individuals are consenting participants in the NIMH Repository and Genomics Resource. The Colorado Multiple Institutional Review Board approved this research.

### Assessment of symptoms and age of onset

All phenotypic data were acquired from the diagnostic interview for genetic studies (DIGS), a scheduled interview designed as part of the NIMH Genetics Initiative.^37^ The DIGS include guidelines for diagnosing disease and contains sections on multiple schizophrenia phenotypes related to the DSM-IV and ICD-10, including positive and negative symptom scales (designed to reflect definitions in ref. 38).

Positive symptom severity in this study was assessed using the scale for the assessment of positive symptoms (SAPS), a past-month measure of positive symptoms. Severity was estimated by adding the global rating scores for each of the SAPS subscales in the DIGS: global hallucinations, global delusions, global bizarre behavior and global positive formal thought disorder. The severity of each of these symptoms is rated from zero (absent) to five (severe). The total possible SAPS score, representing ‘severe' scores for each global symptom category, is 20.

Negative symptom severity was assessed using the scale for the assessment of negative symptoms (SANS), a past-month measure of negative symptoms. Severity was estimated by adding the global rating scores for each of the SANS subscales in the DIGS: global affective flattening, global alogia, global avolition/apathy, global anhedonia/asociality and global attention (deficit). As above, each symptom is rated using a scale from zero (not present) to five (severe). The total possible SANS score, representing ‘severe' scores for each global symptom category, is 25.

Age of onset is the best estimate of age of schizophrenia diagnosis, using data from the DIGS, the Family Interview for Genetic Studies and medical records. This was determined through coordination of clinical records and corroborating family interviews. As age of onset in this population spanned a broad range, for interaction tests age was subdivided into groups (see Statistical analysis section below).

### Genotyping

CON1 and HLS1 copy numbers were assayed using droplet digital PCR as described in ref. 22. Briefly, target DNA, BioRad mastermix, and primers and probes to the test sequence (CON1 or HLS1) and a reference sequence of known copy number in the genome (RPP30), were mixed and loaded into oil droplets that were then subject to a standard thermocyling protocol. A copy number estimate was assigned based on target and reference assay status in each droplet. Merged estimates from three runs per sample were then used to create a consensus copy number assignment for each individual. Assays were conducted in a blinded and randomized manner. The reproducibility of the assay was estimated using the intraclass correlation coefficient on samples assayed in triplicate. The intraclass correlation coefficient for CON1 was 0.84, and for HLS1 was 0.74 suggesting a good to excellent level of reproducibility. Finally, it is important to note that the assay yields a genome-wide estimate of total CON1 and total HLS1 copy number.

### Statistical analysis

Regression analyses were used to identify associations between CON1 copy number, HLS1 copy number and disease phenotypic variation, and differences between cases and controls. Sex and age of onset were explored as covariates in all models, and reduced models for CON1 and HLS1 association analyses were developed through backwards selection. Covariates were kept in the final model if they were significantly associated with the phenotype being examined. SAPS and SANS scores were also explored as covariates as previous evidence suggests that DUF1220 copy number may be associated with highly specific phenotype characteristics (for example, with social deficits in autism^22,23^). Further, the SAPS and SANS scores in this population are correlated with one another (*R*^2^=0.26 *P*<0.001) and the adjustment therefore allows for a more precise phenotypic assessment. Residual diagnostics suggested that the data met assumptions for linear regression. R version 3.0.2 (http://cran.r-project.org/) was used for all analyses.

Covariates that remained were age of onset and negative severity in both the CON1 and HLS1 versus positive severity analyses, and positive severity and sex in both the CON1 and HLS1 versus negative severity analyses. When examining age of onset, the best-fit model included HLS1, CON1 and sex in a single model. Interactions by sex were explored, and owing to information suggesting different etiologies based on age of onset we explored interactions by age of onset group. Age of onset was divided into childhood onset, defined as onset before age 13, adolescent onset defined as 13−19, and adult onset defined as age 20 and older. Interactions of DUF1220 clade by age group and sex were explored in all analyses. Where group-specific effects were suggested by the interaction term (*P*⩽0.15), further analyses were stratified by sex or age.

We also stratified the schizophrenic population into groups with predominantly positive or predominantly negative symptoms, similar to previous studies.^34, 35, 36^ This was performed based on aforementioned work suggesting that the two symptom types are associated with unique neurobiologic and physiologic features and the purported similarity between negative symptoms and symptoms of autism. The negative symptom group was defined as individuals with high negative symptom score (⩾15) and low or moderate positive symptom scores (<12). The positive symptom group was defined as individuals with high positive symptom scores (⩾12) and low or moderate negative symptom scores (<15). Cutoffs were based on the 75th percentile of severity scores for each category. Analysis of variance was then used to identify differences among the positive, negative, ASD and control populations.

Three individuals in this population were recorded with an exceptionally young age of onset (below age 4). To ensure that results are robust to an age of onset recording error in these individuals, a sensitivity analysis was conducted. This analysis compared results including these young individuals to results with them removed. No appreciable differences were observed and results are presented for the entire population.

## Results

### Population characteristics

In the schizophrenic population, CON1 copy number ranged from 47 to 81 and HLS1 copy number ranges from 146 to 261 copies in the diploid genome. In the control population, CON1 copy number ranged from 54 to 79 and HLS1 copy number from 148 to 283. In the ASD population, CON1 copy number ranged from 54 to 78 copies, and HLS1 from 124 to 257 copies. The copy numbers of both DUF1220 subtypes exhibit Gaussian distributions in each population (Supplementary Table 1). In the schizophrenic group, but not control or ASD group, males had a slightly lower CON1 copy number on average than females (*b*=−1.0, s.e.=0.4, *P*=0.01). Further, the individuals with autism used were predominantly multiplex (96.4%), male (79.8%), on average had an IQ of 100 as assessed by the Raven Progressive Matrices, and had an average age of 10.6. All individuals with autism met criteria for diagnosis as assessed by the Autism Diagnostic Interview-Revised and were classified as autism or autism spectrum by the Autism Diagnostic Observation Schedule.

### Schizophrenia positive symptoms

Both CON1 and HLS1 copy number decreases exhibited a significant linear association with increased positive symptom severity in the schizophrenic population (Table 1 and Table 2). We detected a notable interaction by age group for both CON1 and HLS1 versus positive symptoms that suggested a stronger effect in individuals with adult age of onset (interaction *P*-value: CON1 by adult versus adolescent *P*=0.023, HLS1 by adult versus adolescent *P*=0.06). Given the interaction, stratified analyses were preformed and the stratified populations' characteristics are presented in Table 2. No associations were found in the childhood onset group (*N*=37, and no associations were found in the adolescent-onset group (*N*=221; Table 1 and Table 2). In the adult group, meanwhile, a single copy decrease in CON1 was associated with a 0.16 increase in positive symptom score (*P*=0.00155; Table 1), and a single copy decrease in HLS1 was associated with a 0.038 increase in positive symptom score (*P*=0.00361; Table 2). Thus as copy number of either DUF1220 subtype decreased, positive symptoms became progressively worse, a trend uniquely significant in individuals of adult onset.

### Schizophrenia negative symptoms

In the CON1 versus negative symptom analysis, suggestive interactions by sex were observed (*P*=0.15) and additional analyses were stratified with population characteristics presented in Supplementary Table 3. Testing in male (*N*=307), and female (*N*=302) subgroups demonstrated that CON1 copy number increase is positively and linearly associated with an increase in negative symptom score specifically in males, such that for each single copy increase in CON1, SANS score increases by 0.16 points *P*=0.0327, (Table 1). In females no association was found (*b*=0.02, s.e.=0.06, *P*=0.70). These results demonstrate that as CON1 copy number increases, negative symptoms become progressively worse uniquely in males. This association between CON1 copy number and negative symptom severity is similar to findings previously reported for autism severity.^22, 23^

### Comparisons among individuals with schizophrenia, ASD and healthy controls

When examining schizophrenia subgroups (Supplementary Table 4), the positive symptom-dominant group (*n*=66) exhibited significantly reduced CON1 copy number compared with controls (*P*=0.02), the negative symptom-dominant group (*n*=52; *P*=0.007), and a suggestive difference compared with the ASD group (*P*=0.07) (Table 3). Interaction tests in the CON1 analysis again suggested an interaction by sex (*P*=0.08). Follow-up tests showed that the CON1 copy number difference was driven by differences seen among males in each group (Table 4), and that the CON1 copy number difference between predominantly positive symptom schizophrenia and ASD is significant in males (*P*=0.012).

When examining HLS1 copy number differences, an overall test with controls did not demonstrate significant differences at the group level (F-test *P*=0.13), although there was a notable difference between the two schizophrenia subgroups, which demonstrated a nearly eight-copy decrease in HLS1 in the positive symptom group compared with the negative symptom group (*P*=0.036, data not shown).

## Discussion

### Summary of key findings

To the best of our knowledge, this is the first direct investigation of the role of DUF1220 CNV in schizophrenia. We identified associations between DUF1220 copy number and both positive and negative symptoms of schizophrenia, and differences in copy number among schizophrenia symptom subgroups, controls and individuals with autism. Specifically, schizophrenic individuals with predominantly positive symptoms had significantly reduced CON1 copy number compared with schizophrenics with predominantly negative symptoms, and also compared with controls. This difference was most pronounced in the male population, which also exhibited a significant reduction in CON1 copy number in predominantly positive symptom schizophrenics compared with ASD. In the schizophrenic population as a whole, decreasing CON1 and HLS1 DUF1220 subtype copy number were each linearly associated with increasing positive symptoms as measured by the SAPS. Increasing CON1 also exhibited a significant association with increased negative symptoms in males.

The findings that DUF1220 copy number is linearly associated with schizophrenia symptoms are notably similar to our previous findings in two studies of ASD, in which DUF1220 subtype copy number was linearly associated with disease severity in individuals with ASD. These results are relevant to refining the prevailing theories regarding the relationship between autism and schizophrenia. As detailed in the Introduction, these theories include an overlapping phenotype theory, a subtype theory and a diametric opposites theory.

### Negative symptoms

Considering negative symptomology in schizophrenia, the association between increasing CON1 copy number and increasing negative symptoms in males lend support to a model in which negative symptoms of schizophrenia and symptoms of autism are similar and share genetic risk factors, in this case CON1 copy number increase. This is supported by our previous findings that increased CON1 copy number is associated with increased severity of autism symptoms, notably asociality, communicative deficits and internalization, that overlap substantially with the negative symptoms of schizophrenia. Furthermore, the apparent sex effect in which CON1 exhibits a more pronounced association with negative symptoms in males also suggests etiologic overlap and is supported by previous studies of gender effects in these disorders. Autism and schizophrenia both exhibit gender bias such that they are diagnosed more frequently in males versus females^39, 40^ and negative symptoms are commonly found to be more severe in males with schizophrenia,^41^ a finding also observed in this study.

### Positive symptoms

In contrast, though, is the highly significant linear association between both CON1 and HLS1 copy number and positive symptoms. Here, decreased copy number of either DUF1220 subtype is strongly associated with increased positive symptom severity. The association between CON1 copy number and positive symptoms lends support instead to the theory that at least some components of autism and schizophrenia represent diametric opposites of one another. The varying strength of association with positive symptoms in different age groups is also an intriguing finding. The strongest association between DUF1220 copy number and positive symptoms is in the adult-onset population. In contrast, there is a lack of association with positive symptoms in both the child-onset and adolescent-onset populations. Although this may be due to the small sample size of the child-onset population (*N*<40), the lack of an appreciable effect size in individuals of adolescent onset (*b*=0.003, *N*=221) suggests that even with a much larger study no effect would be observed. This may indicate that different genetic factors are involved at different stages of development, or exert differential effects at different developmental time points. Previous work has also found that positive symptoms increase with age,^42^ suggesting that genetic influences on positive symptoms may exert the greatest effect in older schizophrenic individuals.

Further, the opposing association with CON1 in negative and positive symptoms, where increased copies of CON1 are associated with worsening negative symptoms in males and improving positive symptoms, may support findings suggesting that positive and negative symptoms are in part etiologically independent.^43, 44^ However, the normal distribution of DUF1220 copy number in the human population, ranging from 250 to 350 haploid copies, and variation in the location, identity and nature of specific DUF1220 copy number changes in the genome, have the potential to confer a broad phenotypic spectrum. The extremes of these changes are associated with divergent phenotypic variations (for example, positive and negative symptom types) and as a result may appear to have varying etiologic significance.

### Importance of symptom features: schizophrenia subgrouping elucidates differences compared with controls and ASD

A key finding in this study is that if the schizophrenic population is stratified into subgroups defined by symptom features, notable DUF1220-related genetic differences among subgroups and between subgroups and controls become apparent. Here we show that individuals with predominantly positive symptoms have a significantly reduced CON1 copy number compared to controls, and to schizophrenic individuals with predominantly negative symptoms. In addition, males with predominantly positive symptoms have significantly reduced CON1 copy number compared with males with ASD. This suggests that treating all individuals with schizophrenia as having an identical disease may mask genetic and genomic differences that would otherwise be obvious in certain subpopulations with unique disease features.

### DUF1220 and the autism−schizophrenia relationship

**Figure 1:**
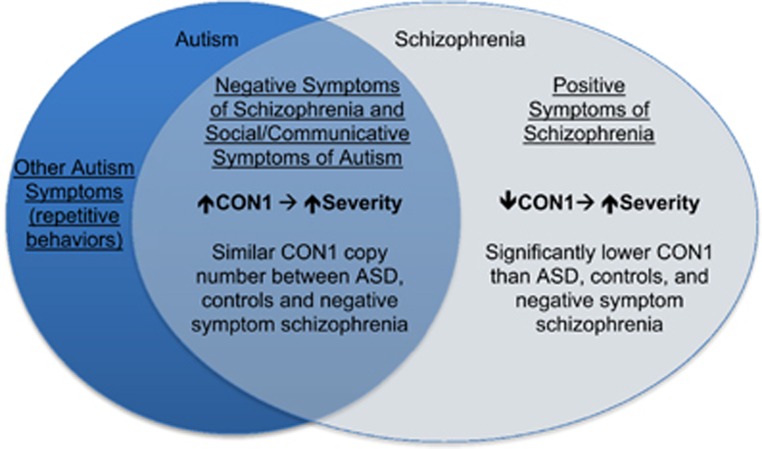
DUF1220 copy number associations support autism and schizophrenia being related disorders. CON1 associations with negative symptoms in schizophrenic males, and with social/communicative symptoms in ASD, suggest these phenotypes overlap between the disorders. The inverse association between CON1 and positive symptoms suggest that positive symptoms could be considered as an opposing phenotype to ASD. ASD, autism spectrum disorder.

Taken together, the opposing associations between CON1 copy number and schizophrenia positive (*P*=0.0068) and negative symptoms in males (*P*=0.0327) suggest that CON1 copy number increase may generally influence specific symptoms that are shared between autism and schizophrenia, while in the schizophrenic neurodevelopmental context CON1 and HLS1 may affect different pathways that contribute to positive symptoms. This is further supported by our findings demonstrating that CON1 copy number is similar between schizophrenics with predominantly negative symptoms and autistic individuals. Meanwhile, CON1 is significantly different between schizophrenic males with predominantly positive symptoms and those with autism (*P*=0.012). Figure 1 highlights these findings, where negative symptoms of schizophrenia and social and communicative symptoms of autism are overlapping based on our genomic evidence and previous phenotypic evidence, but positive symptoms of schizophrenia are isolated, exhibiting divergent genomic results than those seen in autism or for negative phenotypes of schizophrenia. The associations that exhibit gender-specific effects are also notable given the aforementioned gender bias in both disorders, the predominance of negative symptoms in males, the unique male risk effects seen in neonates of schizophrenic mothers, and the increased risk of autism in offspring of women with schizophrenia and other severe mental illness.^45^

We suggest, then, that our results support the previously proposed overlapping theory of autism and schizophrenia when discussing negative symptoms, but that a diametric opposites theory is more likely when considering schizophrenia positive symptoms. It is plausible that schizophrenia itself should be considered as an disease continuum, where one end of the continuum features predominantly negative symptoms and clearly overlaps with autism, while the other end of the continuum features predominantly positive symptoms and exhibits no overlap with autism (although may overlap with other diseases featuring similar symptoms). These results suggest that further research into the etiology of autism and schizophrenia should emphasize shared and unshared symptoms in addition to simple disease diagnosis.

### Concordance with previous CNV findings

It should also be noted that large 1q21.1 deletions are enriched in individuals with schizophrenia and reciprocal duplications enriched in individuals with autism.^26^ Many DUF1220 domains are interspersed throughout the 1q21.1 region within these disease-associated CNVs.^9^ Therefore, changes in DUF1220 copy number may in part explain associations between 1q21 CNV and autistic and schizophrenic phenotypes. For example, our results demonstrating that decreases in HLS1 and CON1 copy number are linearly associated with increasing positive severity is consistent with 1q21 deletions being enriched in individuals with schizophrenia. This would be further supported if individuals with schizophrenia and 1q21 deletions had uniquely severe positive symptoms. Although there is no literature available on this point, there have been reports that, compared with negative symptoms, positive symptoms represent a significantly greater proportion of the genetic liability associated with schizophrenia.^17^ This is consistent with 1q21 deletions being more frequently found in schizophrenia compared with autism and with our finding that DUF1220 copy number decrease is linearly associated with increased positive (but not negative) symptom severity.

The observation that 1q21 duplications are more common in individuals with autism, but seen in both conditions, suggest that 1q21 duplications may be associated with a broadly negative/autistic symptom phenotype. Our results demonstrating that increased CON1 copy number is associated with both increased autism severity and increased negative symptom severity in schizophrenia is consistent with this theory and suggests an overlapping etiology underlying negative-type symptomology in the two diseases.

### Mechanistic explanations

Previous studies of DUF1220 in neuropathology and neural stem cells suggest potential mechanisms that may mediate these associations. Increased CON1 copy number has previously been associated with increased total gray matter volume, with the most notable associations seen for the frontal surface area and volume, temporal volume and thickness, and parietal thickness.^8^ These gray matter regions have each been implicated in schizophrenia neuropathology, with decreases seen in schizophrenic individuals in multiple MRI-based studies.^33, 46^ Studies examining the frontal lobe specifically have noted decreased volume, decreased cortical neuron number and decreased metabolism.^47^ Given these findings, it is noteworthy that DUF1220 copy number increase is associated with increased cortical neuron number and that increased DUF1220 expression induces increased neuronal stem cell proliferation in cultured cells.^48^ Thus, there may be a neuroproliferative mechanism related to DUF1220 copy number that is in turn associated with modification of symptom severity in the schizophrenic (and autistic) neurodevelopmental environment.

### Strengths and limitations

Our finding that there was no obvious difference in subtype copy number between the schizophrenic population as a whole and the control population may be misleading and warrants some clarification. The assay we used to measure DUF1220 subtype copy number, droplet digital PCR, provides an assessment of global (genome-wide) copy number and, as a result, does not clarify which copies in the genome are changing. In the case of CON1, which is found in virtually all *NBPF* genes, this means that CON1 copy number across ~20 different genes is being measured in each reaction. Importantly, it is possible that some DUF1220-related changes may lead to disease while others may be relatively innocuous, an occurrence that would not be detectable by our assay method. Thus it is possible that the nature of the CON1 copy number change (that is, which specific copies are inserted in, or deleted from, the genome) is critical to whether disease occurs or not. Such technical limitations of droplet digital PCR should be addressed through further research that uses technologies such as Irys and long-read sequencing that are capable of following individual copies of DUF1220 subtypes in the genome.

The above limitations notwithstanding, the results reported here have significant implications for the genetics of autism and schizophrenia and for how these disorders are classified. The data suggest that: (1) DUF1220 copy number may have a particularly important role in the severity of symptoms of schizophrenia; (2) the symptom severity in autism is associated in the same fashion with CON1 as negative symptom severity is in schizophrenia, suggesting that these may be partially overlapping disorders that are mechanistically and genetically related; (3) DUF1220 copy number is reduced in schizophrenia individuals with predominantly positive symptoms compared with controls, autistic individuals, and schizophrenic individuals with predominantly negative symptoms, highlighting the importance of considering the confounding effects of disease stratification on genotype−phenotype associations; and (4) the linear, DNA-dosage dependent nature of the associations we report here, and previously for autism, is consistent with the possibility that DUF1220 copy number is contributing to a continuum of severity for both disorders, in which copy number gain increases autism and schizophrenia negative symptom severity, while loss increases schizophrenia positive symptom severity. Having previously linked DUF1220 dosage to the evolutionary increase in human brain size, we have now implicated DUF1220 copy number in the symptoms of both autism and schizophrenia, supporting a new direction for research into the etiology and genetics of these pervasive and debilitating disorders. Finally, these findings lend further support to the view that autism and schizophrenia are harmful by-products of human brain evolution, resulting in part from the rapid and extreme evolutionary increase in DUF1220 copy number in the human genome.

## Figures and Tables

**Table 1:** CON1 associations with schizophrenia features

*Phenotype measure*	*Beta*	*s.e.*	P*-value*
SAPS: total population^a^	−0.097	0.039	**0.013**
SAPS: childhood onset	0.15	0.15	0.22
SAPS: adolescent onset	0.003	0.06	0.70
SAPS: adult onset	−0.16	0.05	**0.00155**
SANS: males^b^	0.16	0.07	**0.0327**

Abbreviations: SANS, scale for the assessment of negative symptoms; SAPS, scale for the assessment of positive symptoms.

Results from regression analyses of DUF1220 subtype CON1 copy number versus positive symptom score (SAPS), negative symptom score (SANS) stratified by age of onset. The stratified population description is available in Supplementary Table 2. *P*-values meeting statistical significance (<0.05) are in bold.

a CON1 versus SAPS model includes SANS and age as covariates; age-specific models include SANS as a covariate.

b CON1 versus SANS model includes SAPS as covariate.

**Table 2:** HLS1 associations with schizophrenia features

*Phenotype measure*	*Beta*	*s.e.*	P*-value*
SAPS: total population^a^	−0.022	0.01	**0.0227**
SAPS: childhood onset	−0.03	0.03	0.28
SAPS: adolescent onset	0.003	0.017	0.83
SAPS: adult onset	−0.038	0.013	**0.00361**
SANS: males^b^	0.027	0.017	0.119

Abbreviations: SANS, scale for the assessment of negative symptoms; SAPS, scale for the assessment of positive symptoms.

Results from regression analyses of DUF1220 subtype HLS1 copy number versus positive symptom score (SAPS), negative symptom score (SANS) stratified by age of onset. The stratified population description is available in Supplementary Table 2.

*P*-values meeting statistical significance (<0.05) are in bold.

a HLS1 versus SAPS model includes SANS and age as covariates; age-specific models include SANS as a covariate.

b HLS1 versus SANS model includes SAPS as covariate.

**Table 3:** Comparison of CON1 copy number in schizophrenia positive symptom group to ASD, control and schizophrenia negative symptom groups

*Comparison group*	*CON1 compared with positive symptom schizophrenia group*	*s.e.*	P*-value*
ASD	+1.23 CON1 copies	0.677	0.071
Control	+1.62 CON1 copies	0.715	**0.0237**
Negative symptom schizophrenia	+2.34 CON1 copies	0.861	**0.0068**

Abbreviation: ASD, autism spectrum disorder.

Positive symptom schizophrenia group as reference (mean=64.39) versus copy number of comparison groups. The stratified population description is available in Supplementary Table 3. *P*-values meeting statistical significance (<0.05) are in bold.

**Table 4:** Comparison of CON1 in male subgroups to schizophrenia positive symptom male group

*Comparison group*	*CON1 compared with positive symptom schizophrenia group*	*s.e.*	P*-value*
ASD	+2.09 CON1 copies	0.829	**0.0124**
Control	+2.41 CON1 copies	0.938	**0.0107**
Negative symptom schizophrenia	+2.81 CON1 copies	1.09	**0.0106**

Abbreviation: ASD, autism spectrum disorder.

Positive symptom schizophrenia male group as reference (mean=65.44, *N*=40) versus copy number of male comparison groups. The stratified population description is available in Supplementary Table 3. *P*-values meeting statistical significance (<0.05) are in bold.
