# Supplementary material for: DUF1220 copy number is associated with schizophrenia risk and severity: implications for understanding autism and schizophrenia as related diseases
Source: Transl Psychiatry. 2015 Dec 15;5(12):e697–. doi: 10.1038/tp.2015.192 (PMC5068589; doi:10.1038/tp.2015.192)
Supplement: Supplementary Tables [file tp2015192x1.docx]

**Supplementary Table 1: Schizophrenia (n=609***)***, control (n=120), and autistic (n=168) population characteristics**

| Schizophrenia | Characteristic | Min | 1^st^ quartile | Mean | Median | 3^rd^ quartile | Max |
| --- | --- | --- | --- | --- | --- | --- | --- |
|  | Age at Onset | 1 | 17 | 21 | 21.6 | 25 | 58 |
|  | SAPS | 0 | 4 | 8 | 8.2 | 12 | 20 |
|  | SANS | 0 | 6 | 10 | 10.5 | 14 | 25 |
|  | CON1 copy number | 47 | 63 | 66 | 66 | 68 | 81 |
|  | HLS1 copy number | 146 | 186 | 199 | 199 | 211 | 261 |
| Controls | Characteristic | Min | 1st quartile | Mean | Median | 3rd quartile | Max |
|  | CON1 copy number | 54 | 63 | 66 | 66 | 69 | 79 |
|  | HLS1 copy number | 148 | 186 | 199 | 199 | 212 | 283 |
| ASD | Characteristic | Min | 1st quartile | Mean | Median | 3rd quartile | Max |
|  | CON1 copy number | 54 | 63 | 66 | 66 | 68 | 78 |
|  | HLS1 copy number | 124 | 185 | 196 | 198 | 209 | 257 |

Description of missing data;

Individuals with schizophrenia;16 missing age, 40 missing SAPS, 73 missing SANS, 11 missing CON1and 11 missing HLS1.

Controls; 1 missing CON1 and 2 missing HLS1

Individuals with asd; 2 missing CON1 and 4 missing HLS1

**Supplementary Table 2: Characteristics of age stratified populations of individuals with schizophrenia**

|  | Characteristic | Min | 1^st^ quartile | Mean | Median | 3^rd^ quartile | Max |
| --- | --- | --- | --- | --- | --- | --- | --- |
| Child Onset | Age | 1.0 | 5.0 | 7.8 | 7.0 | 11.0 | 12.0 |
| N = 37 | SAPS | 1.0 | 7.0 | 9.3 | 9.5 | 12.3 | 15.0 |
|  | SANS | 0.0 | 5.0 | 9.5 | 10.0 | 14.0 | 22.0 |
|  | CON1 copy number | 53.9 | 64.2 | 65.8 | 65.8 | 67.4 | 73.3 |
|  | HLS1 copy number | 145.7 | 177.1 | 193.6 | 195.8 | 204.5 | 231.8 |
|  | Characteristic | Min | 1^st^ quartile | Mean | Median | 3^rd^ quartile | Max |
| Adolescent | Age | 13.0 | 16.0 | 16.9 | 17.0 | 18.0 | 19.0 |
| N = 221 | SAPS | 0 | 4.3 | 8.5 | 8.0 | 12.0 | 19 |
|  | SANS | 0 | 7.0 | 11.3 | 11.0 | 16.0 | 25.0 |
|  | CON1 copy number | 51.8 | 62.9 | 66.0 | 65.9 | 69.1 | 80.7 |
|  | HLS1 copy number | 152.3 | 186.5 | 199.3 | 199.1 | 209.8 | 258.8 |
|  | Characteristic | Min | 1^st^ quartile | Mean | Median | 3^rd^ quartile | Max |
| Adults | Age | 20.0 | 21.0 | 26.3 | 25.0 | 30.0 | 58.0 |
| N = 335 | SAPS | 0 | 4.0 | 7.9 | 8.0 | 11.0 | 20.0 |
|  | SANS | 0 | 5.0 | 10.1 | 10.0 | 14.0 | 25.0 |
|  | CON1 copy number | 47.0 | 62.0 | 65.2 | 65.6 | 68.2 | 81.5 |
|  | HLS1 copy number | 146.0 | 185.8 | 199.9 | 199.1 | 211.9 | 260.6 |

Description of missing data;

Child Onset; 1 missing SAPS, 2 missing CON1.

Adolescent Onset;15 missing SAPS, 25 missing SANS, 2 missing CON1 and 3 missing HLS1.

Adult Onset;19 missing SAPS, 43 missing SANS, 7 missing CON1 and 8 missing HLS1.

**Supplementary Table 3: Characteristics of sex stratified populations of individuals with schizophrenia**

|  | Characteristic | Min | 1^st^ quartile | Mean | Median | 3^rd^ quartile | Max |
| --- | --- | --- | --- | --- | --- | --- | --- |
| Males | Age | 1 | 17.0 | 20.9 | 20.0 | 24.0 | 48.0 |
| N = 307 | SAPS | 0 | 5 | 8.9 | 9.0 | 12.5 | 25.0 |
|  | SANS | 0 | 7.0 | 11.4 | 11.0 | 15.3 | 25.0 |
|  | CON1 copy number | 47.0 | 62.1 | 65.0 | 65.5 | 67.7 | 81.3 |
|  | HLS1 copy number | 145.7 | 184.4 | 198.4 | 198.0 | 209.0 | 251.7 |
|  | Characteristic | Min | 1^st^ quartile | Mean | Median | 3^rd^ quartile | Max |
| Females | Age | 1 | 17.0 | 21.6 | 21.0 | 25.0 | 58.0 |
| N = 302 | SAPS | 0 | 4.0 | 8.2 | 8.0 | 11.0 | 20.0 |
|  | SANS | 0 | 5.0 | 9.6 | 9.0 | 14.0 | 25.0 |
|  | CON1 copy number | 48.9 | 63.3 | 66.0 | 66.2 | 69.3 | 81.5 |
|  | HLS1 copy number | 150.8 | 187.0 | 200.2 | 199.7 | 212.2 | 260.6 |

Description of missing data;

Males; 11 missing age, 28 missing SAPS, 27 missing SANS, 7 missing CON1, 4 missing HLS.

Females; 5 missing age, 12 missing SAPS, 46 missing SANS, 4 missing CON1, 7 missing HLS.

*.*

**Supplementary Table 4: Characteristics of the positive and negative symptom dominant populations of individuals with schizophrenia**

|  | Characteristic | Min | 1^st^ quartile | Mean | Median | 3^rd^ quartile | Max |
| --- | --- | --- | --- | --- | --- | --- | --- |
| Positive Symptom Group | Age | 3.0 | 16.0 | 19.9 | 19.0 | 23.0 | 35.0 |
| N = 66 | SAPS | 12.0 | 13.0 | 14.0 | 14.0 | 15.0 | 17.0 |
|  | SANS | 0.0 | 7.0 | 9.4 | 10.0 | 12.0 | 14.0 |
|  | CON1 copy number | 52.6 | 61.4 | 64.4 | 65.0 | 67.2 | 77.4 |
|  | HLS1 copy number | 146.0 | 182.3 | 195.9 | 196.0 | 206.8 | 241.0 |
|  | Characteristic | Min | 1^st^ quartile | Mean | Median | 3^rd^ quartile | Max |
| Male  Positive Symptom  Group | Age | 3 | 17.0 | 19.9 | 19.0 | 23.5 | 35.0 |
| N = 40 | SAPS | 12.0 | 13.0 | 14.0 | 14.0 | 15.0 | 17.0 |
|  | SANS | 0.0 | 6.8 | 9.2 | 10.0 | 12.0 | 14.0 |
|  | CON1 copy number | 53.5 | 61.0 | 63.7 | 63.6 | 66.2 | 77.4 |
|  | HLS1 copy number | 146.0 | 182.3 | 195.8 | 199.1 | 208.1 | 241.0 |
|  | Characteristic | Min | 1^st^ quartile | Mean | Median | 3^rd^ quartile | Max |
| Male  Negative Symptom  Group | Age | 5.0 | 16.0 | 19.8 | 18.5 | 23.5 | 35.0 |
| N = 32 | SAPS | 0.0 | 4.8 | 7.0 | 8.0 | 10.0 | 11 |
|  | SANS | 15.0 | 16.0 | 18.1 | 18.0 | 19.0 | 24.0 |
|  | CON1 copy number | 57.7 | 64.0 | 66.5 | 66.7 | 69.6 | 73.8 |
|  | HLS1 copy number | 176.2 | 189.2 | 203.1 | 200.6 | 212.2 | 250.0 |

Description of missing data;

Positive Symptom Group; 1 missing age, 1 missing CON1, 2 missing HLS.

Male Positive Symptom Group; 1 missing age.

Male Negative Symptom Group; none missing.
